# Obesity, antioxidants and negative symptom improvement in first-episode schizophrenia patients treated with risperidone

**DOI:** 10.1038/s41537-023-00346-z

**Published:** 2023-03-23

**Authors:** Zhiyong Gao, Meihong Xiu, Jiahong Liu, Fengchun Wu, Xiang-Yang Zhang

**Affiliations:** 1 The Affiliated Kangning Hospital of Wenzhou Medical University, Zhejiang Provincial Clinical Research Center for Mental Disorder, Wenzhou, China; 2 Peking University HuiLongGuan Clinical Medical School, Beijing HuiLongGuan Hospital, Beijing, China; 3 Department of Psychiatry, The Affiliated Brain Hospital of Guangzhou Medical University, Guangzhou, China; 4 Guangdong Engineering Technology Research Center for Translational Medicine of Mental Disorders, Guangzhou, China; 5 CAS Key Laboratory of Mental Health, Institute of Psychology, Beijing, China

**Keywords:** Schizophrenia, Biomarkers

## Abstract

Negative symptoms remain a main therapeutic challenge in patients with schizophrenia (SZ). Obesity is associated with more severe negative symptoms after the first episode of psychosis. Oxidative stress caused by an impaired antioxidant defense system is involved in the pathophysiology of SZ. Yet, it is unclear regarding the role of obesity and antioxidants in negative symptom improvements in SZ. Therefore, this longitudinal study was designed to assess the impact of obesity on antioxidant defenses and negative symptom improvements in first-episode SZ patients. A total of 241 medication-naive and first-episode patients with SZ were treated with risperidone for 3 months. Outcome measures including symptoms, body weight, and total antioxidant status (TAS) levels were measured at baseline and the end of the third month. We found that after 12 weeks of treatment with risperidone, the body weight increased and clinical symptoms significantly improved. Baseline body mass index (BMI) was negatively correlated with negative symptom improvement after treatment and an increase in TAS was negatively associated with an increase in BMI only in the high BMI group. More importantly, the TAS × BMI interaction at baseline was an independent predictor of negative symptom improvement. Our longitudinal study indicates that the improvement in negative symptoms by risperidone was associated with baseline BMI and TAS levels in patients with SZ. Baseline BMI and TAS may be a predictor for negative improvement in SZ patients after risperidone treatment.

## Introduction

Schizophrenia (SZ) is a severe mental illness characterized by persistent or relapsing episodes of positive symptoms and negative symptoms^1^. Antipsychotics are recommended as the first-line medication for treating SZ and have been reported in clinical trials to be effective in improving symptoms and behaviors related to SZ. Positive symptoms can be effectively treated by available antipsychotics, modern therapy, and psychiatric support^2^. It has been shown that about 60% of SZ patients can return home after recovery from their first episode and ~50% can return to work^3,4^. However, ~30% remains severely disabled by their condition and 10% must be hospitalized^5,6^.

The percentage of obesity in patients with SZ is significantly higher than in the general population^7,8^. According to recent literature, 40–60% of individuals with SZ are reported to be overweight or obese^9,10^. Recent meta-analyses of patients with medication-naïve and first-episode psychosis (MNFE) have revealed increased insulin resistance and impaired glucose tolerance relative to healthy controls^11^, although earlier studies have shown that patients with first-episode psychosis were diagnosed with much less diabetes than chronic patients on antipsychotics^12^. Moreover, studies reported rapid weight gain (>7%), usually within 6–8 weeks after treatments with antipsychotics^13^. Presumably, patients with SZ may be prone to manifest pre-diabetes, which emphasizes the need for early monitoring of overweight/obesity in first-episode SZ.

There is accumulating evidence to reveal an association between obesity, metabolic parameters, or the changes and clinical symptoms in patients with MNFE^14,15^. For example, studies have reported that body mass index (BMI) and other metabolic parameters, including the homeostasis model assessment of insulin resistance (HOMA-IR), and hemoglobin A1C (HbA1c) were associated with negative symptoms in SZ patients^16,17^. Even multiple studies showed that weight gain is related to decreased general psychopathology in patients with SZ treated with second-generation antipsychotics^18,19^. Some recent evidence revealed that weight gain is an important prognostic marker of treatment response to antipsychotics^20,21^, suggesting that body weight and weight gain are important issues in therapeutic benefits in SZ.

On the other hand, studies have also focused on the essential role of oxidative stress in the pathogenesis of obesity, metabolic disorders, and SZ^22,23^. Glutathione peroxidase, catalase, and superoxide dismutase are the main enzymatic antioxidants in the cells^24,25^. Vitamins A and C, tocopherol, glutathione, uric acid, albumin, and bilirubin are important non-enzymatic antioxidants^26,27^. These antioxidants help detoxify harmful reactive oxygen species to protect from ROS-induced damage to proteins, DNA, and mitochondrial membranes^27^. Alterations in serum or plasma levels or activities of antioxidants have been reported in patients with obesity or SZ^28–33^. Total antioxidant status is an important indicator of the additive antioxidant effect in vivo, which is measured via ferric reducing antioxidant potential (FRAP).

Atypical and typical antipsychotics have an impact on antioxidant defense system in patients with SZ^34–37^. Antipsychotic medication may induce oxidative stress, which further influences the turnover of catecholamines and suppresses the activity of antioxidant enzymes. There is clear evidence for the different effects of typical and atypical antipsychotics in regulating oxidative stress and antioxidant defense systems^38–40^. Risperidone is one of the most widely prescribed atypical antipsychotics used in the treatment of SZ, for both acute and long-term medication^41^. It is a benzisoxazole derivative and has a strong binding affinity for D_2_ and 5-HT receptors^42^. Animal studies provide strong evidence for the regulation of risperidone in the redox system. It has antioxidant effects by increasing the antioxidant defenses, such as regulating glutathione levels in C6 astroglial cell model^43^. Administration of risperidone can restore the brain glutathione levels and decrease total antioxidant capacity in rats induced by perinatal phencyclidine^44,45^. In addition, risperidone treatment significantly upregulates antioxidant enzyme activities in patients with SZ^46–48^.

Atypical antipsychotics have been reported to be linked to rapid weight gains and obesity in the initial period after antipsychotic medications in SZ patients^49^. Based on the close relationship between antipsychotic treatment, obesity, and redox regulation in patients with SZ, the aim of this study was to investigate the effect of antioxidants and BMI on the clinical outcome of risperidone in patients with SZ. We hypothesized that the association between TAS levels and clinical symptom improvement following risperidone treatment would be different between the low and high BMI groups and that the interaction between TAS and BMI would predict the response to risperidone in SZ patients. Notably, to minimize the potential effects of types and accumulative doses of antipsychotics, physical-health comorbidity, and duration of illness, only MNFE patients with SZ were recruited in the present study.

## Materials and methods

### Participants

A total of 241 MNFE patients (128 males/113 females) diagnosed with SZ by SCID-IV, between the age of 16 and 45 years were recruited from Beijing Huilongguan Hospital and Henan Zhumadian psychiatric hospitals in China. Eligibility inclusion/exclusion criteria were described in our prior studies^30,50^, and detailed criteria were described in the supplementary materials. In brief, inclusion criteria included both sexes, illness duration ≤5 years, no previous treatment with psychotropic medicines or cumulative use of antipsychotics ≤14 days and the clinical global impression (GCI) of 4 or over. The patients had a mean ± SD age of 27.6 ± 9.2 years, a mean BMI of 21.3 ± 3.4 kg/m^2^, a mean onset age of 26.1 ± 9.2 years and an average illness duration of 1.5 ± 1.3 years. Of the 241 patients, sixty-six patients were smokers (66/241, 27.4%) and there was no difference in smoking rates between BMI subgroups. The average severity of the illness assessed by the Positive and Negative Syndrome Scale (PANSS) was 76.0 ± 17.4.

The study protocol was approved by the Ethics Committees of Beijing Huilongguan Hospital, and written informed consent was obtained from each patient.

### Study overview

All recruited SZ patients received a flexible dose (4–6 mg/day) of oral risperidone for 3 months. The study consisted of three visits conducted by experienced psychiatrists including a questionnaire survey, clinical assessment scales, and blood sampling on day 1 (visit 1, screening), day 1 (visit 2, baseline assessment), and at the third month or after early discontinuation of risperidone (visit 3, post-treatment assessment).

### Clinical evaluation and TAS measurements

The clinical symptom was assessed by six experienced clinicians using the PANSS^51^. Before the PANSS rating, the raters participated in comprehensive training. After training, the inter-rater reliability was assessed by comparing the rating of PANSS total score for the same patient assessed by six raters and was analyzed using intraclass correlation coefficients (ICC)^52^. A high ICC of PANSS total score was achieved (PANSS-ICC > 0.8). The outcome measures were respectively assessed at baseline and 3-month follow-up. The reduction in PANSS total score or its subscores was calculated by the changes in the total score or subscores from baseline to 3-month follow-up.

According to the obesity criteria^53^, our patients were classified into the low BMI group (BMI ≤ 24 kg/m^2^) and the high BMI group (BMI > 24 kg/m^2^). Overweight/obese participants were included in the high BMI group and the remaining participants constituted the low BMI group. Underweight participants also belonged to the low BMI group. The increase in BMI was calculated by the change in the BMI from baseline to 3-month follow-up.

Plasma TAS levels of all patients were measured at baseline and follow-up. Details of the methods were described in the supplementary materials.

### Statistical analysis

For patients who discontinued the medication after 2 months, the last observation carried forward (LOCF) was used. For patients who discontinued the medication before 2 months, their clinical data were not included in the following analyses. Baseline demographic characteristics were compared between the low-BMI group and high-BMI group using analysis of variance (ANOVA) and *X*^2^ tests. A repeated-measure ANOVA was used to analyze the different changes in PANSS total score and its subscale scores and TAS levels between the low-BMI group and high-BMI group from baseline to the LOCF endpoint at the third month. Pearson’s product moment correlation was performed to investigate the association between symptom improvements and baseline BMI or changes in BMI in the low-BMI group and high-BMI group, respectively. Linear regression analyses were performed to determine the relative contribution of BMI, TAS levels, and other demographic variables to the variance in clinical symptom improvement after treatment.

The data were analyzed using IBM SPSS software (version 22.0, Chicago, IL), and statistical significance thresholds were determined at *P*-value < 0.05.

## Results

### Demographic data and clinical data in patients at baseline

At baseline, patients were classified into two groups according to their BMI values: the low BMI group (*n* = 207) and the high BMI group (*n* = 34). There was a significant difference in age between the low BMI and high BMI groups (*p* < 0.05) (Table 1). Correlation analysis showed that BMI at baseline was associated with age (*r* = 0.25, *p* < 0.001), age at onset (*r* = 0.21, *p* = 0.001), and negative symptoms in SZ patients (*r* = −0.13, *p* = 0.039).

**Table 1:** Demographic and clinical characteristics of patients in the high BMI and low BMI groups.

Variable	Patients		*p* value
	High BMI group (*n* = 34)	Low BMI group (*n* = 207)	
Sex (M/F)	19/15	109/98	0.85
Age (ys)	30.8 ± 8.3	27.1 ± 9.3	0.03
Education (ys)	10.0 ± 4.0	8.9 ± 3.9	0.15
Onset age (ys)	28.7 ± 8.7	25.6 ± 9.2	0.07
TAS (U/ml)	194.9 ± 64.3	227.9 ± 67.8	0.008

*BMI* body mass index; *ys* years.

### BMI and clinical symptom improvements after treatment

After treatment with risperidone for 12 weeks, weight and BMI were significantly increased compared to baseline values (weight: 2.7 ± 3.8 kg; BMI: 1.0 ± 1.3 kg/m^2^, all *p* < 0.05). Moreover, PANSS total score or its subscores were also significantly lower after treatment (all *p* < 0.01) (Table 2). Repeated-measure ANOVA showed no significant difference in the improvements in clinical symptoms after treatment with risperidone between high and low BMI subgroups (all *p* > 0.05) (Table 2). A significant difference in the increase in weight was observed between low BMI and high BMI groups (3.1 ± 3.8 vs 0.4 ± 3.0, *t* = −4.2, *p* < 0.001).

**Table 2:** Comparisons of clinical symptoms before and after 12 weeks of risperidone monotherapy between the low BMI group and high BMI group.

	Baseline		12-week follow-up		Effect		
	Low BMI group	High BMI group	Low BMI group	High BMI group	Time F(p)	Group F(p)	Interaction F(p)
**Clinical symptoms**							
P score	22.0 ± 6.8	22.8 ± 5.3	11.9 ± 4.7	10.9 ± 3.2	298.6 (<0.001)	0.01 (0.91)	2.0 (0.16)
N score	18.9 ± 7.2	18.1 ± 6.2	14.1 ± 5.6	14.6 ± 5.6	44.1 (<0.001)	0.01 (0.91)	1.1 (0.30)
G score	35.7 ± 10.3	37.0 ± 8.9	24.9 ± 6.0	24.5 ± 6.8	149.4 (<0.001)	0.1 (0.75)	0.8 (0.36)
Total score	76.4 ± 18.7	78.0 ± 14.9	50.9 ± 13.2	49.9 ± 12.2	207.2 (<0.001)	0.02 (0.90)	0.5 (0.50)

In addition, we found that the baseline BMI was negatively associated with the increase in BMI (*r* = −0.38, *p* < 0.001). Further subgroup analysis by baseline BMI showed that there was no significant association between baseline BMI and improvements in positive and negative symptoms and general psychology in the low BMI group. Whereas in the high BMI group, a significant inverse association between baseline BMI and improvement in negative symptoms was observed (*r* = −0.44, *p* = 0.016). Moreover, in the high BMI group, an increase in BMI correlated with improvements in general psychopathology (*r* = 0.44, *p* = 0.021) and PANSS total score (*r* = 0.38, *p* = 0.046), but not in the low BMI group (all *p* > 0.05).

### TAS levels and clinical symptom improvements after treatment

After treatment, TAS levels were significantly increased relative to baseline levels (215.3 ± 66.6 vs 266.4 ± 105.5, *p* < 0.05) (increase: 51.1, 95% confidence interval [CI]: 38.0–64.2). Repeated-measure ANOVA showed that there was no significant difference in the increases in TAS levels after treatment between high and low BMI subgroups (*F* = 0.5, *p* > 0.05).

Correlation analyses showed that there was no significant association between the increase in TAS levels and the improvements in clinical symptoms in the low BMI and high BMI groups (all *p* > 0.05). However, we found that in the high BMI group, the increases in TAS levels were negatively associated with the increases in BMI (*r* = −0.46, *p* = 0.014), but not in the low BMI group (*p* > 0.05).

### Interaction effect of TAS levels and BMI on the clinical symptom improvements after treatment

We also found significant associations between TAS levels and BMI in patients at baseline (*r* = −0.18, *p* = 0.006) and 12-week follow-up (*r* = −0.21, *p* = 0.004). Multiple regression analysis found that an interaction effect of BMI and TAS levels was significantly associated with negative symptoms at baseline (*p* < 0.05). Furthermore, multiple regression analysis showed that the TAS levels × BMI interaction was an independent predictor for the improvement in the negative symptoms in the patients with an adjusted *R*^2^ = 0.04 (*β* = −0.15, *t* = −2.1, *p* = 0.035), after controlling for age, gender, and education years.

## Discussion

We found that (1) baseline BMI was correlated with the improvement in negative symptoms after 12 weeks of treatment with risperidone only in the high BMI group, (2) an increase in TAS was negatively correlated with an increase in BMI after treatment only in high BMI group, and (3) the TAS × BMI interaction at baseline was an independent predictor of negative symptom improvement.

This study found that baseline BMI was inversely related to negative symptom improvements only in the overweight/obesity MDFE patients with SZ, but not in patients with normal weight. MDFE patients with higher baseline BMI showed less improvement in negative symptoms after risperidone treatment. Negative symptoms of SZ refer to deficits in certain functions common to most people, such as facial expressions, emotional responses, and joy or motivation^54^. It is a core component of SZ and is linked with poor outcomes in patients with SZ^55–57^. Negative symptoms remain an unmet treatment challenge. In the CATIE study, one of the largest clinical trials in SZ, negative symptoms were found to be common in SZ patients (40%)^58^. In addition, in a meta-analysis of placebo controlled clinical trials of atypical antipsychotic drugs (*n* = 7450), it was reported that negative symptoms presented after 6-week treatment in 1/3 of patients actively participating in treatment^59^. Our findings show that for those patients who were overweight or obese, weight gain needed to be effectively managed to obtain greater negative symptom improvements. The burden of overweight/obesity is both a physical and a psychological problem. An obesity intervention program for MNFE patients with SZ may improve the psychological status of patients. Whereas for the patients with normal weight, fluctuations in body weight may not be correlated with improvements in negative symptoms.

Another finding of this study was that risperidone treatment significantly increased BMI and TAS levels in patients with SZ. In addition, BMI gain after treatment with risperidone was negatively associated with TAS increase in overweight/obese SZ patients. Namely, the more the patient gained weight, the less the increase in TAS levels, which is consistent with our expectations. In line with our findings, animal model studies have also shown that antioxidative enzyme activities were reduced in obese mice following long-term administration of antipsychotics and weight gain^60^. Many previous clinical studies have investigated the association between overweight or obesity and antioxidants, and recent evidence supports an inverse intrinsic relationship between obesity and antioxidant defense system parameters^61–63^. In SZ, there is also some evidence supporting that overweight/obesity induced by antipsychotics is related to redox system biomarkers. Furthermore, it has been reported that the combination of antipsychotics with antioxidants, e.g., extraction of ginkgo biloba (EGb47) improved clinical symptoms in SZ patients with higher baseline BMI^64^. However, we cannot draw a conclusion on the causal relationship between risperidone-induced weight gain and TAS changes based on the current design.

We further found that the interrelationship between baseline BMI and TAS levels was an important predictor of improvements in negative symptoms after treatment with risperidone in SZ. Redox dysregulation, sequent oxidative stress, and metabolic abnormalities have been investigated for many years and are well-established in SZ^65–71^. Cumulative studies have shown that negative symptoms also were associated with antioxidant activities and overweight/obesity^72^. Furthermore, substantial evidence supports that risperidone has an impact on antioxidant enzyme activities and affects body weight^73,74^, which may be further involved in mild to moderate improvement of negative symptoms. Thus, our findings were in line with previously published literature but were incremental with respect to antioxidants and obesity in the mechanism of symptom amelioration with risperidone. Altogether, the most interesting finding of this study was that the interrelationship between BMI and antioxidant defenses at the onset stage of SZ may be a valuable predictor of treatment response to risperidone.

Several limitations should be noted in this study. First, this was a non-experimental study, and as such we were unable to arrive at causal conclusions between overweight/obesity, antioxidant defenses and symptom improvements from prospective observational data using this approach. Second, the follow-up time points for symptom assessment and blood sampling after risperidone monotherapy were limited. Outcome measures were obtained at only two time points (baseline and three months). Third, in this study, the dose of oral risperidone administered to each patient depended on the symptoms judged by the doctor, however, we did not record the detailed dose for each patient. Therefore, we did not add the dose of risperidone as a covariate to eliminate its potential influences on antioxidants and body weight. Forth, the negative symptomatology was not assessed with a specific scale as the Brief Negative Symptom Scale (BNSS) or the Clinical Assessment Interview for Negative Symptoms (CAINS) in this study.

In summary, there was a negative correlation between increases in BMI and TAS levels after risperidone treatment in the high-BMI group. In addition, improvement in negative symptoms in SZ patients was associated with BMI at baseline, suggesting that patients with overweight/obesity during the onset phase of SZ need more attention. For non-obese patients at the onset, there was no correlation between BMI, TAS, and improvements in clinical symptoms. However, considering the small number of SZ patients with overweight or obesity, further studies with a large sample size of SZ patients and a randomized double-blind clinical trial design are necessary to elucidate the mechanisms underlying this interrelationship between obesity, antioxidant defense system, and negative symptoms of SZ patients.

## Supplementary information

SUPPLEMENTAL MATERIAL
