# Supplementary material for: Obesity, antioxidants and negative symptom improvement in first-episode schizophrenia patients treated with risperidone
Source: Schizophrenia (Heidelb). 2023 Mar 23;9(1):17. doi: 10.1038/s41537-023-00346-z (PMC10033648; doi:10.1038/s41537-023-00346-z)
Supplement: Supplementary file 1 — SUPPLEMENTAL MATERIAL [file 41537_2023_346_MOESM1_ESM.docx]

1. ***Inclusion and exclusion criteria***

Patients should meet the following inclusion criteria: a SCID diagnosis; informed consent; males or females; age 16-45 years; Han nationality; course of disease less than 5 years; no previous treatment with psychotropic medicines or cumulative use of antipsychotic drugs less than 14 days; the clinical global impression (GCI) of 4 or over; no major medical comorbidities; treatment with risperidone was not contraindicated; and without abuse or substance dependence except tobacco.

The patients were screened by 4 psychiatrists, including a SCID interview and physical examination. Diagnoses were made for each patient at baseline and at follow-up by two independent experienced psychiatrists. The exclusion criteria included 1) with a history of treatment with psychotropic drugs (e.g., mood stabilizers, anti-anxiety drugs, antidepressants, or antipsychotics) for more than 2 weeks; 2) with physical diseases (i.e., cancer, diabetes, and hypertension); 3) with a history of head trauma with residual effects, neurological disorders, and uncontrolled major medical conditions; 4) had any history of alcohol or drug abuse (aside from tobacco); 5) had ongoing infections, allergies, or a past history of autoimmune disorders; 6) were pregnant or breastfeeding, 7) took over-the-counter antioxidants; and 8) were unable to provide signed consent forms.

1. ***Plasma TAS measurements***

The TAS of fasting plasma was measured as ferric reducing antioxidant potential (FRAP) by using a commercially available kit in all subject samples. In this assay, antioxidants are evaluated as reductants of Fe3+ to Fe2+, which is chelated by TPTZ to form a Fe2+–TPTZ complex absorbing at 593 nm. 10 μl plasma samples diluted 1:4 in water were mixed in a 96-well plate with 300 μl of a reagent solution containing 1.7 mM FeCl3 and 0.8 mM TPTZ in 300 mM sodium acetate, pH 3.6. The samples were incubated for 15 min at 37 °C, and the absorbance at 593 nm was
recorded using the Multiskan microplate reader (FlowLabs, McLean, VA, USA). The activity was expressed as Units per milliliter plasma (U/ml).
